# Evaluation of Etiological Causes and Incidence of Pancytopenia: A Case Series from a Tertiary Care Center

**DOI:** 10.7759/cureus.79485

**Published:** 2025-02-22

**Authors:** İlker Çordan, Tufan Tukek

**Affiliations:** 1 Department of Internal Medicine, Division of Endocrinology, University of Health Sciences, Konya City Hospital, Konya, TUR; 2 Department of Internal Medicine, Istanbul Faculty of Medicine, Istanbul University, İstanbul, TUR

**Keywords:** aplastic anemia, brucellosis, hematological malignancies, hypersplenism, megaloblastic anemia, myelodysplastic syndrome, pancytopenia, systemic lupus erythematosus, vitamin b12 deficiency

## Abstract

Background and aim

Pancytopenia is a clinical condition characterized by the simultaneous reduction of red blood cells, white blood cells, and platelets. It generally arises from conditions associated with bone marrow dysfunction or increased peripheral consumption of blood cells. Understanding the regional etiological models of pancytopenia is of paramount importance for optimizing diagnostic and therapeutic strategies specific to the local population. This study aimed to identify the etiological causes and examine the distribution of incidence rates in patients presenting with pancytopenia in Istanbul and cases referred from across Turkey.

Methods

This retrospective study was conducted between January 2008 and November 2010 in the general internal medicine clinic of a tertiary care training and research hospital serving a large population. The study included 112 adult patients newly diagnosed with pancytopenia who had complete clinical and laboratory data. Data were obtained through detailed laboratory analyses, diagnostic tests, and clinical evaluations.

Results

Of the 112 patients, the female-to-male ratio was 1.8:1, and the mean age was 56.5 years, with the most common age group being 70-79 years (19.6%, n=22). Pancytopenia was most frequently caused by vitamin B12 deficiency and hypersplenism (20.5%, n=23). These were followed by hematological malignancies (acute leukemia, lymphoma, and multiple myeloma) at 16% (n=18), infections and myelodysplastic syndromes at 10.7% (n=12), drug-induced pancytopenia at 5.4% (n=6), systemic lupus erythematosus at 4.5% (n=5), metastatic solid organ tumors at 3.6% (n=4), and aplastic anemia at 1.8% (n=2).

Conclusion

This study demonstrated that the most common causes of pancytopenia in the evaluated patient group in Istanbul and cases referred from across Turkey were vitamin B12 deficiency and hypersplenism. This finding underscores the importance of prioritizing systemic conditions over hematological malignancies in the diagnostic process. Accurate identification of underlying causes and improved patient management require a structured diagnostic approach involving thorough clinical evaluations and targeted investigations tailored to regional characteristics.

## Introduction

Pancytopenia is a clinical syndrome characterized by the simultaneous reduction of red blood cells (RBCs), white blood cells (WBCs), and platelets, often signifying an underlying disorder such as bone marrow dysfunction or excessive peripheral consumption of blood cells [1]. The clinical spectrum ranges from asymptomatic cases to severe complications that can rapidly become life-threatening [2]. This variability necessitates careful clinical evaluation and a systematic approach to diagnosis and treatment.

Patients with pancytopenia typically present with symptoms such as pallor, fatigue, bleeding tendencies, bruising, or susceptibility to infections. However, in some cases, pancytopenia is incidentally detected during routine blood tests. The etiological causes of pancytopenia exhibit significant variability influenced by factors such as age, sex, ethnicity, socioeconomic conditions, cultural habits, and endemic diseases [3]. While regional variations exist, common causes of pancytopenia include nutritional deficiencies, hypersplenism, infections, hematological malignancies, and aplastic anemia [4].

Identifying the underlying causes is crucial for ensuring accurate treatment and assessing prognosis. The diagnostic process fundamentally relies on comprehensive evaluations, including medical history, physical examination, and laboratory testing. In cases where non-invasive methods prove insufficient, advanced procedures such as bone marrow biopsy play a pivotal role. Treatment involves managing symptoms and addressing underlying causes, with advanced interventions such as blood transfusion, stem cell therapy, or bone marrow transplantation utilized when necessary [5]. Evaluating variations in diagnostic and treatment approaches by considering regional characteristics may increase the effectiveness of patient management. Despite the significant effect of epidemiological factors on the clinical presentation of pancytopenia, studies examining the etiological distribution of pancytopenia in Istanbul and cases referred from across Turkey remain notably limited.

This study aimed to identify the most common underlying causes of pancytopenia in patients presenting with this condition and to fill the existing knowledge gap by providing comprehensive data on its distribution. Conducted in the internal medicine clinic of a high-volume tertiary healthcare institution that serves as a national referral center for suspected cases of pancytopenia, this study sought to elucidate both regional and national epidemiological patterns of the condition. Furthermore, the findings are expected to contribute to improving early diagnosis and optimizing diagnostic and treatment processes for clinicians by providing data on the causes of pancytopenia.

## Materials and methods

This study was conducted between January 2008 and November 2010 in the Department of Internal Medicine at Vakıf Gureba Training and Research Hospital. All adult patients confirmed to have pancytopenia, with complete clinical and laboratory data, and meeting the inclusion criteria were enrolled in the study through outpatient clinics or emergency services.

Selection criteria

For the diagnosis of pancytopenia, the threshold values for hemogram parameters were defined as white blood cell count ≤4.5x10³/µL, hemoglobin <13 g/dL for men, hemoglobin <12 g/dL for women, and platelet count ≤150x10³/µL. Adult patients newly diagnosed with pancytopenia showing reductions in all three blood cell lineages were included in the study. Excluded from the study were patients with previously identified causes of pancytopenia who were undergoing treatment, individuals who had recently received blood transfusions, patients undergoing chemotherapy or radiotherapy, those with congenital disorders such as thalassemia or sickle cell anemia, pregnant women, and patients under 16 years of age. Furthermore, individuals with incomplete medical records, laboratory results, or patient histories were excluded from the study.

Laboratory analysis

Biochemical analyses were performed in the central laboratory of Vakıf Gureba Training and Research Hospital. Venous blood samples were collected in tubes containing ethylenediaminetetraacetic acid (Franklin Lakes, NJ: Vacutainer; Becton, Dickinson and Company) for hemogram parameter measurements and analyzed within two hours. Complete blood count results were obtained using an Abbott Cell Dyn3700 analyzer (Gurnee, IL: Abbott Laboratories). It was verified that all internal and external quality control results remained within acceptable ranges throughout the analytical process. The reference ranges for laboratory parameters were based on institutional laboratory standards.

Diagnostic evaluation process and variables

Detailed patient histories, including age, sex, occupation, place of residence, medication use, and alcohol consumption, were documented in detail. Based on anamnesis and physical examination findings, further diagnostic evaluations were performed to confirm potential and differential diagnoses. Non-invasive laboratory tests and imaging methods were prioritized during the diagnostic process.

As part of routine clinical management, the patients underwent basic hematological tests such as complete blood count, peripheral blood smear, and reticulocyte count. To assess nutritional anemia, serum iron, serum ferritin, vitamin B12, and folic acid levels were measured. Additional tests were performed on selected patients in accordance with clinical suspicion. In cases of clinical suspicion, thyroid and liver function tests, anterior pituitary hormones, anti-nuclear antibodies, and anti-dsDNA autoantibodies were examined. To rule out or confirm infectious causes, advanced tests such as hepatitis and HIV serology, the Rose Bengal test and standard tube agglutination test for brucellosis, the Widal test for Salmonella infection, a thick blood smear for malaria diagnosis, and blood cultures were performed. When the condition was suspected to have a bone marrow origin, bone marrow aspiration and biopsy were conducted. Abdominal ultrasonography was performed to evaluate underlying abdominal pathologies.

Due to the retrospective nature of the study, no additional written consent was obtained from the patients; however, during hospital admission, all patients routinely provided written consent for the use of information obtained during diagnostic and treatment procedures for scientific purposes. The study did not include any interventions or experimental procedures beyond routine clinical care. The researchers recorded findings obtained solely from standard diagnostic and treatment procedures without influencing clinical management.

Statistical analysis

The collected data were organized using Microsoft Excel (Redmond, WA: Microsoft) and analyzed with IBM SPSS Statistics version 20.0 for Windows (Armonk, NY: IBM Corp.). As the study did not include a comparative analysis, descriptive statistics were employed to examine the distribution of age groups, sex, and etiological causes. The normality of continuous variables was assessed using the Shapiro-Wilk test. Categorical variables were presented as frequencies and percentages, whereas numerical variables were reported as mean±standard deviation or median (Q1-Q3) values, depending on the data distribution.

## Results

**Figure 1:**
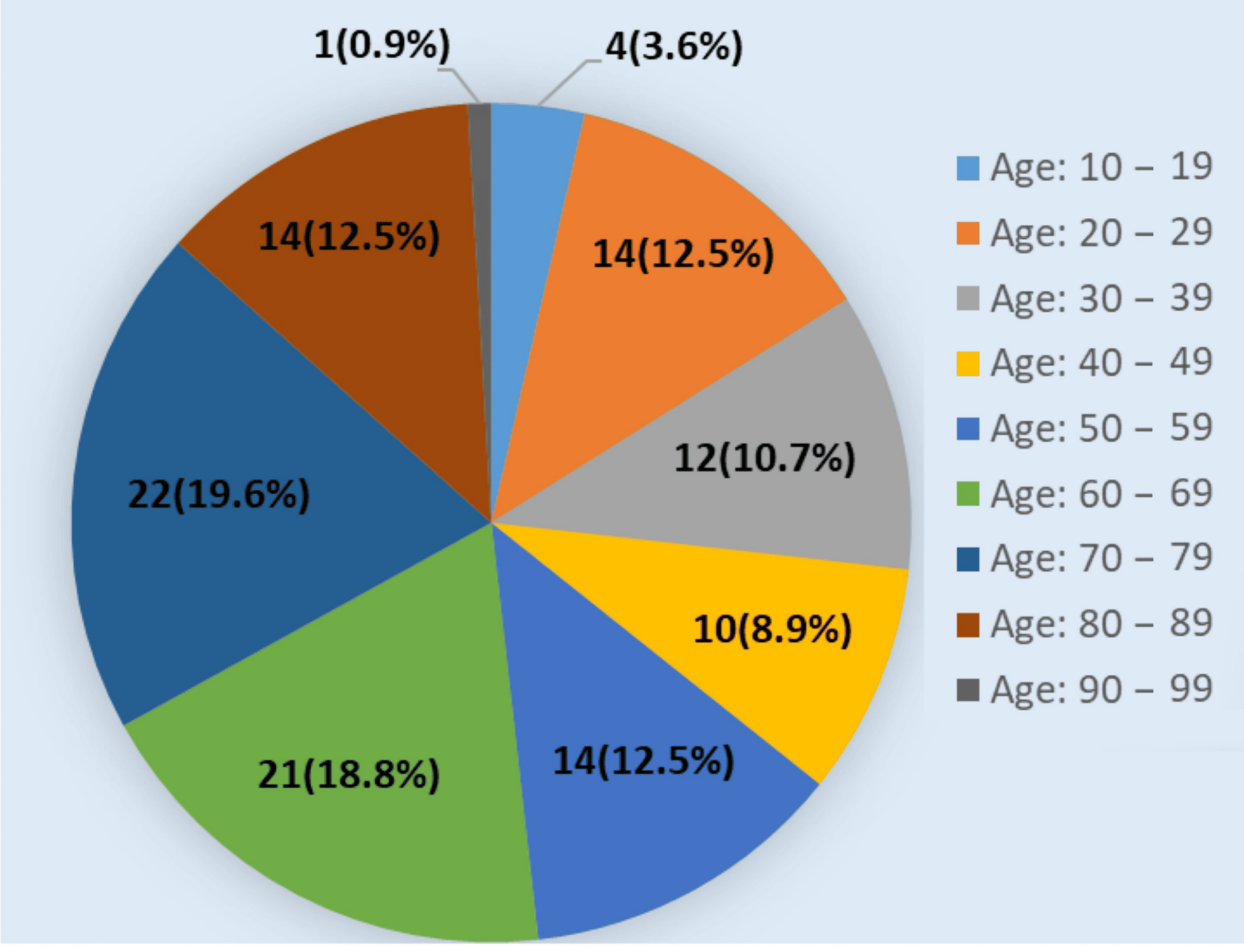
Distribution of patients by age (in years) groups, representing the proportion and number of patients in each decade.

Of the 112 patients included in the study, 72 were female (64.3%) and 40 were male (35.7%), resulting in a female-to-male ratio of 1.8:1. The patients’ ages ranged from 16 to 94 years, with a mean age of 56.50±20.7 years. The most common age group was 70-79 years (n=22, 19.6%), followed by 60-69 years (n=21, 18.8%) and 20-29 years and 80-89 years (each n=14, 12.5%). The demographic characteristics of the patients are presented in Table 1, and the age distribution by decades is presented in Figure 1.

**Table 1:** Demographic and laboratory characteristics of the patients. ^a^The values are presented as mean±standard deviation for continuous variables and as n (%) for categorical variables. WBC: white blood cell count; RBC: red blood cell count Note: Empty cells are indicated with a hyphen (-).

Variables	n (%)^a^	Reference values	Units
Age (mean±SD) in years	56.50±20.72	-	-
Female	72 (64.3)	-	-
Male	40 (35.7)	-	-
WBC count	3.05±0.99	4.5-10.0	x10³/µL
RBC count	2.94±0.88	4.1-5.9	x10³/mm³
Hemoglobin	8.12±2.49	12.5-16.0 (female), 13.5-18.0 (male)	g/dL
Hematocrit	25.72±6.28	37-47 (female), 40-52 (male)	%
Platelet count	85.83±37.11	150-400	x10³/µL
Severe anemia	21 (18.7)	≤7	g/dL
Severe neutropenia	7 (6.3)	≤500	mm³
Severe thrombocytopenia	7 (6.3)	≤20,000	x10³/µL

**Figure 2:**
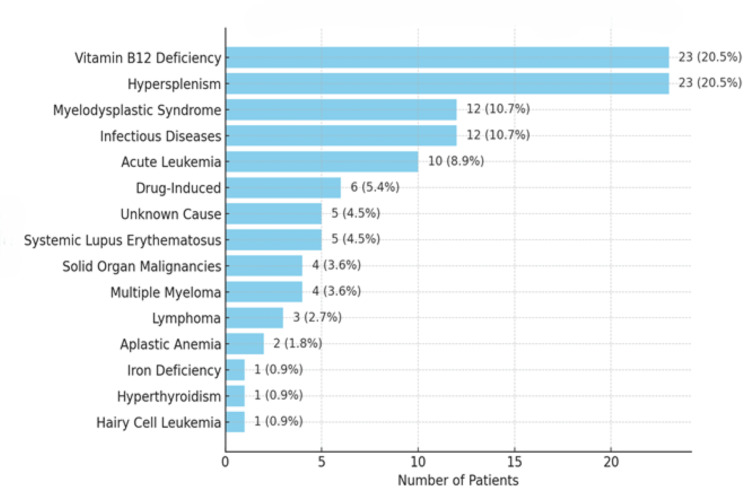
Etiological distribution of pancytopenia among patients.

In the evaluation of patients’ hematological parameters, the mean values for leukocyte, erythrocyte, hemoglobin, hematocrit, and platelet counts were determined. The analysis revealed severe thrombocytopenia (platelet count <20,000x10³/µL) in seven patients (6.3%), severe anemia (hemoglobin <7 g/dL) in 21 patients (18.9), and severe neutropenia (neutrophil count <500/mm³) in seven patients (6.3%). The details of these hematological findings are presented in Table 1. The most common etiological causes were vitamin B12 deficiency (n=23, 20.5%) and hypersplenism (n=23, 20.5%). The etiological causes and their frequencies among all patients with pancytopenia are summarized in Figure 2.

Among the 23 patients with vitamin B12 deficiency, 12 were male (52.2%) and 11 were female (47.8%), with a mean age of 65.2±15.5 years. Vitamin B12 deficiency was most commonly observed in the 50-59 years and 80-89 years age groups (n=6, 26.0% each).

Of the 23 patients with pancytopenia due to hypersplenism, 19 were female (82.6%) and four were male (17.4%). Liver cirrhosis was the cause in the majority of cases (n=18, 78.2%). Among patients with liver cirrhosis, 14 were female (77.7%) and four were male (22.3%). Other causes of hypersplenism included idiopathic hypersplenism, non-cirrhotic portal hypertension, and hereditary spherocytosis. The mean age of patients with liver cirrhosis was 59.7±15.6 years, with the highest proportion observed in the 60-69 years age group (n=8, 44.4%). The causes and frequencies of hypersplenism-related pancytopenia are summarized in Table 2.

**Table 2:** Distribution of hypersplenism-related pancytopenia. N/A: not available

Etiology	n (%)	Male, n (%)	Female, n (%)
Liver cirrhosis	18 (78.3)	4 (17.4)	14 (60.9)
Idiopathic hypersplenism	2 (8.7)	N/A	2 (8.7)
Non-cirrhotic portal hypertension	2 (8.7)	N/A	2 (8.7)
Hereditary spherocytosis	1 (4.3)	N/A	1 (4.3)
Total	23 (100.0)	4 (17.4)	19 (82.6)

Hematological malignancies were identified in 18 patients (16.0%). Among 10 patients diagnosed with acute leukemia (8.9%), five were female and five were male. In females, two had acute myeloid leukemia (AML) and three had acute lymphoblastic leukemia (ALL), while in males, three had AML and two had ALL. The mean age was 55.4 years for those with ALL and 47.4 years for those with AML. Multiple myeloma was identified in four patients (3.6%) (two females, two males) with a mean age of 76.2 years. Of the three patients diagnosed with lymphoma, one had Hodgkin lymphoma and two had non-Hodgkin lymphoma. In addition, hairy cell leukemia was identified in a 63-year-old male patient.

Myelodysplastic syndromes caused pancytopenia in 12 patients (10.7%), with five males (41.6%) and seven females (58.4%). The mean age was 69.9±15.3 years, with the highest proportion in the 70-89 years age group (33.3%).

Pancytopenia due to infectious diseases was observed in 12 patients (10.7%), including five males (41.6%) and seven females (58.4%). The most frequent cause was sepsis, detected in six patients, with ages ranging from 36 to 83 years. Causes of sepsis included pneumonia (n=3), infective endocarditis (n=1), and unknown causes (n=2). Additionally, brucellosis was detected in three patients and *Salmonella paratyphi* infection in one patient. The details of infectious causes are given in Table 3.

**Table 3:** Distribution of infectious diseases causing pancytopenia. N/A: not available

Etiology	n (%)	Male, n (%)	Female, n (%)
Sepsis	6 (50.0)	4 (33.3)	2 (16.7)
Brucellosis	3 (25.0)	1 (8.3)	2 (16.7)
Salmonella paratyphi	1 (8.3)	N/A	1 (8.3)
Viral infections	2 (16.7)	N/A	2 (16.7)
Total	12 (100.0)	5 (41.7)	7 (58.3)

Pancytopenia due to systemic lupus erythematosus (SLE) (n=5, 4.5%) and drug use (n=6, 5.4%) occurred exclusively in female patients. All patients diagnosed with SLE were in their reproductive period. Drugs responsible for drug-induced pancytopenia included linezolid, clonazepam, interferon-alpha, hydroxyurea, azathioprine, and carbamazepine. Two patients (1.8%) were diagnosed with aplastic anemia (a 20-year-old female and a 48-year-old male). Additionally, pancytopenia due to hyperthyroidism was identified in a 61-year-old female and iron deficiency in a 21-year-old female.

Five patients (4.5%) remained undiagnosed. Of these patients, three died during the diagnostic process, and two were discharged upon their own request. A total of eight patients (7.1%) died during their hospital stays. Causes of death included sepsis (n=2; ages 67 and 87 years), metastatic prostate carcinoma (n=1; age 53 years), ALL (n=1; age 61 years), AML (n=1; age 55 years), and unknown causes (n=3; ages 18, 53, and 84 years). Among these, an 18-year-old female patient exhibited blasts consistent with acute leukemia in the peripheral smear, but due to the absence of a biopsy before her death, she was classified as undiagnosed.

## Discussion

Pancytopenia is not a disease in itself but rather an indicator of an underlying clinical condition, and in some cases, it may serve as the initial manifestation of rapidly progressive and life-threatening disorders such as hematological malignancies (e.g., acute leukemia and myelodysplastic syndromes) or severe infections (e.g., sepsis) [6]. Therefore, developing a comprehensive diagnostic algorithm and adopting a multidisciplinary approach in cases where an initial diagnosis cannot be established is of critical importance. Our study explored the most common causes of pancytopenia in Turkey and shed light on regional variations in these causes. These findings can contribute to the establishment of swift and effective diagnostic processes in clinical practice.

In our study, the two most frequent causes of pancytopenia were identified as vitamin B12 deficiency and hypersplenism, accounting for nearly half of all cases. The majority of hypersplenism cases were associated with liver cirrhosis, indicating that nutritional deficiencies, viral hepatitis, and chronic liver diseases play a significant role in the etiology of pancytopenia in Istanbul and cases referred from across Turkey. In addition, infectious diseases, particularly endemic illnesses such as brucellosis and Salmonella infections, have noteworthy effects on pancytopenia, although they contribute to a smaller number of cases. On the other hand, primary hematologic diseases originating in the bone marrow (e.g., acute leukemia, lymphoma, and multiple myeloma) were observed less frequently in our study.

Vitamin B12 deficiency can result from various causes, including inadequate dietary intake, eating disorders, alcoholism, intrinsic factor deficiency, and malabsorption [7,8]. Pancytopenia due to vitamin B12 deficiency is observed in 5% of individuals with this deficiency, with prevalence rates reaching up to 74% in low socioeconomic conditions [9,2]. Both our study and similar research conducted in Turkey identified vitamin B12 deficiency as one of the most common causes of pancytopenia [10,11]. The diagnosis is supported by findings such as macrocytic red blood cells and hypersegmented neutrophils in the peripheral smear, along with neurological symptoms, ataxia, and a positive Romberg test. Observing a reticulocyte response on days 4-7 after appropriate replacement therapy and examining hypercellular findings in the bone marrow, if necessary, can strengthen the diagnosis [12]. Early diagnosis and treatment can prevent unnecessary costly tests and serious hematologic and neurologic complications.

Hypersplenism is a significant clinical condition causing pancytopenia through sequestration and destruction of blood cells due to splenomegaly. In our study, hypersplenism was identified as one of the frequent causes of pancytopenia, a result consistent with the literature [10]. However, there are regional variations in the distribution of factors leading to hypersplenism, and this situation directly influences treatment approaches. In Turkey, hypersplenism often results from chronic liver diseases associated with hepatitis B and C infections. Although its frequency has declined with routine vaccination programs, hypersplenism remains the most common cause [13]. In Western countries, alcohol consumption plays a more dominant role in the etiology of liver cirrhosis compared to other factors [14,15]. In contrast, endemic infections such as malaria and visceral leishmaniasis are prevalent causes of hypersplenism in developing countries [16-19]. Simple liver function tests, e.g., albumin, prothrombin time, and international normalized ratio, as well as imaging methods such as ultrasonography, can provide a rapid and effective assessment.

Infectious diseases hold a significant place in the etiology of pancytopenia, with brucellosis and Crimean-Congo hemorrhagic fever being particularly notable in Turkey [20,21]. Although malaria has been eradicated, it remains an etiological consideration in migrant populations [22]. In our study, brucellosis cases were observed among individuals engaged in livestock farming in Eastern Anatolia, and one patient presented with enteric fever. While pancytopenia cases caused by viral infections (e.g., Parvovirus B19) often resolve spontaneously, severe infections such as sepsis, as observed in our study, are associated with high mortality rates and require multidisciplinary care. Familiarity with local endemic diseases can facilitate diagnosis and enable effective management. Furthermore, simple and economical methods such as thick blood smear, viral serology, and agglutination tests can rapidly diagnose these conditions without requiring complex testing processes.

Primary bone marrow disorders such as myelodysplastic syndrome, hematologic malignancies, and aplastic anemia are among the most common causes of pancytopenia in Western societies, but their frequency was lower in our study [23,24]. Myelodysplastic syndrome is generally a disease of advanced age, and in our study, the majority of patients were aged 70-90 years, supporting this observation. Aplastic anemia was identified at a low rate in our study; however, it is more prevalent in Asian and Far Eastern countries, where it is often linked to environmental factors such as pesticide exposure [25,26].

The frequency of diseases leading to pancytopenia varies according to regional etiological differences as well as patient age and sex [2]. In our study, the female-to-male ratio was found to be higher in favor of women, a finding consistent with the study by Erişmiş et al. [11]. The higher prevalence of hypersplenism, SLE, and drug-induced causes among female patients may have contributed to this distribution. Furthermore, factors such as the relatively young age of our patient population and the study being conducted in an internal medicine clinic, where systemic diseases are predominantly evaluated, may have affected these results. Similar findings were reported in a study by Yokuş and Gedik, which demonstrated that chronic liver diseases were more common in older female patients, whereas drug use and autoimmune diseases were more frequently observed in younger patients [10]. However, studies focusing primarily on hematological disorders have shown that sex distribution may vary across different age groups and geographical regions [27-29]. The fact that all lupus patients in our study were women of reproductive age further supports the role of age and sex as determinants in differential diagnosis.

Drug-induced pancytopenia is a severe adverse effect associated with various medications. Although it is most frequently linked to chemotherapeutic agents that affect bone marrow, other drug classes, including antibiotics, anticonvulsants, antithyroid agents, and non-steroidal anti-inflammatory drugs, can also induce pancytopenia [30]. In our study, cases of drug-induced pancytopenia were predominantly associated with agents used in the treatment of rheumatologic and neurological disorders, with all affected patients being female. This finding underscores the need for a more careful assessment of the effect of certain diseases and their treatments on vulnerable populations. In hematological disorders accompanied by hypersplenism, the use of tyrosine kinase inhibitors has been associated with fatal complications such as spontaneous splenic rupture [31]. This emphasizes the necessity of cautious medication management in high-risk patient groups. Particularly for female patients, closer follow-up and regular hematological monitoring may be crucial for the early detection and prevention of potential complications. These findings highlight the significance of medication use in differential diagnosis, reinforcing the need for taking a detailed patient history and, when necessary, discontinuing suspected drugs and exploring alternative treatment options.

Rarely observed conditions in our study, such as Graves’ disease and iron deficiency anemia, are typically reported as rare case reports in the literature [32,33]. These findings highlight the need for careful evaluation of clinical clues and emphasize the importance of a comprehensive and individualized approach in managing pancytopenia.

Our findings emphasize the importance of considering regionally prevalent diseases, sex, age, underlying chronic conditions, and the medical agents used in their treatment when evaluating the etiology of pancytopenia. This approach may assist clinicians in developing diagnostic strategies and prioritizing relevant laboratory tests. The identification of common and rapidly diagnosable causes such as vitamin B12 deficiency and hypersplenism may facilitate early intervention and improve patient outcomes. To validate these findings and improve their applicability to broader populations, there is a need for prospective, multicenter, and comprehensive studies that are not limited to a specific department.

Among the strengths of this study are its relatively large sample size and the determination of pancytopenia causes in all patients except those who died. Furthermore, the inclusion of the entire adult population allows for a more comprehensive evaluation compared to hematology-based studies, which predominantly focus on hematological malignancies and typically target older age groups. However, the study also has certain limitations. Due to the retrospective design, there is an inherent risk of selection bias and missing data, which may have affected the accuracy and generalizability of the findings. Since the data were obtained from patient records, some clinical information and laboratory parameters may not have been consistently documented. In addition, although the study was conducted in a center serving a broad region, its results may have been influenced by local health issues and demographic factors. Due to the restriction of the study population to patients referred to the internal medicine department, the findings may not fully represent the general population. Moreover, the exclusion of pediatric patients prevents an evaluation of congenital causes of pancytopenia. Lastly, while all laboratory tests were performed in a single accredited laboratory, limitations related to preanalytical factors, such as sample handling, storage conditions, and analytical techniques, may have influenced the results. Prospective studies employing standardized data collection methods in the future can contribute to a more robust validation of our findings.

## Conclusions

Our study demonstrated that the most common etiological causes of pancytopenia in adult patients presenting in Istanbul and cases referred from across Turkey were vitamin B12 deficiency and hypersplenism. This finding underscores the need to prioritize non-hematological causes and chronic diseases over primary bone marrow disorders in internal medicine practice. In addition, the effect of endemic infections on pancytopenia has been noted. Including a broad spectrum of clinical evaluations, our study emphasizes the importance of comprehensive diagnostic approaches and provides valuable data that can guide daily clinical practice. However, broader, multicenter, and prospective studies are needed to improve the generalizability of the results.
